# Demographic, Social, and Economic Factors of Internalizing Problems in Referred and Non-Referred Adolescents

**DOI:** 10.3390/ijerph17145195

**Published:** 2020-07-18

**Authors:** Lucía Antolín-Suárez, Francisco J. Nieto-Casado, Ana Rodríguez-Meirinhos, Alfredo Oliva

**Affiliations:** 1 Departamento de Psicología Evolutiva y de la Educación, Universidad de Sevilla, 41018 Seville, Spain; luciaantolin@us.es (L.A.-S.); oliva@us.es (A.O.); 2 Department of Communication and Education, Universidad Loyola Andalucía, 41704 Dos Hermanas, Seville, Spain; arodriguezm@uloyola.es

**Keywords:** demographic factors, social factors, economic factors, internalizing problems, depressive symptoms, suicidal ideation, adolescence

## Abstract

Depressive symptoms and suicidal ideation are common internalizing problems during adolescence. Numerous studies have explored the role of certain demographic, social, and economic factors in their development in referred or non-referred adolescents, but not simultaneously in both groups. In this study, we examined the association between age, gender, parents’ educational level, and socioeconomic status (SES) and depressive symptoms and suicidal ideation in a referred group (*n* = 211) and a non-referred (*n* = 1401) group of adolescents. We also examined the moderating role that these factors play in the relationships between both internalizing problems. The results showed: higher levels of depressive symptoms and suicidal ideation in the referred group; an increase in both problems during early-to-middle adolescence in the non-referred group; an association between low SES and suicidal ideation in both groups; an association between low father’s education level and depressive symptoms in the non-referred group; and no gender differences in either of these two internalizing problems. The moderation analyses showed that age, in referred adolescents, and SES, in non-referred adolescents, moderated the relationship between depressive symptoms and suicidal ideation. This study contributes to the identification of groups of vulnerable adolescents that could constitute the target populations of preventive programs.

## 1. Introduction

Mental disorders constitute one of the greatest public health problems of our time. Several studies have shown that one out of four people in the world develop mental health problems at some point in their life [1]. Adolescence is a key period during which the incidence of externalizing and internalizing problems increases significantly [2,3,4].

The internalizing problems that most commonly occur during this developmental stage are depressive symptoms and suicidal ideation. Research has found a rapid increase in depressive symptoms and suicidal ideation from early to late adolescence [5,6], with their being occurrence greater in girls than boys [7,8,9]. In addition, depressive symptoms and suicidal ideation have been postulated to be the main predictors of adolescent suicide [10,11,12]. Thus, to identify the causes of these internalizing problems has become an international priority for suicide prevention among youths.

With the focus on the prevention and understanding of these problems, in this study, we explored the association between certain demographic, social, and economic factors and the manifestation of depressive symptoms and suicidal ideation in two populations: referred adolescents, namely outpatients using mental health facilities, and non-referred adolescents, namely community adolescents. Furthermore, we aimed to clarify the moderating role that certain demographic, social, and economic factors may play in the relationship between depressive symptoms and suicidal ideation.

### 1.1. Depressive Symptoms and Suicidal Ideation during Adolescence: Age and Gender Differences

Adolescence is a developmental stage during which individuals are particularly vulnerable to the development of mental health problems [13,14], particularly internalizing problems [15]. Most studies report that the levels of some internalizing problems, such as depressive symptoms and suicidal ideation, increase from early to late adolescence [5,6]. However, other studies have found that the levels of these problems remain stable during this period [16,17]. In early adulthood, the levels of these problems begin to decline for some individuals, while they become chronic for others.

Regarding gender differences, the available evidence also contains discrepancies. Many studies suggest that adolescent girls from referred and non-referred samples experience higher levels of depressive symptoms [18,19,20,21] and suicidal ideation [17,22] than boys. However, there are also studies that have not found gender differences in non-referred samples [23,24,25]. Likewise, other studies conducted simultaneously in referred and non-referred samples have found that levels of depressive symptoms were significantly higher in girls than in boys from the referred group, but that there were no gender differences in the non-referred group [26]. Although these results highlight the need to conduct studies that clarify gender differences based on the type of analyzed sample, few studies to date have simultaneously explored the existence of gender differences in samples of referred and non-referred adolescents.

The comorbidity and stability of depressive symptoms and suicidal ideation have also been studied along with gender differences. In recent decades, ample research with the adolescent population has documented a strong and stable correlation between depressive symptoms and suicidal ideation [27,28]. In addition, both of these internalizing problems can persist over time, affecting mental health during adulthood [1,29,30,31,32]. In fact, both depressive symptoms and suicidal ideation are well-established risk factors of suicide [10,11,12], which constitutes the second most frequent cause of death worldwide in individuals between 10 and 24 years old [33]. The high stability of these manifestations and their association with future health problems could explain the current interest of the scientific community in identifying the factors involved in their development during adolescence [34].

### 1.2. Social and Economic Factors of Depressive Symptoms and Suicidal Ideation

It has been shown that depressive symptoms and suicidal ideation are not determined by a single cause. These complex phenomena have multiple determinants [35,36] and are caused by a conjunction of biological, psychological, social, economic, and cultural factors [37,38].

Regarding social factors, research has focused on the contribution of socioeconomic status and parents’ educational level to depressive symptoms and suicidal ideation in adolescents. These studies indicate that adolescents who come from families with a low socioeconomic status are at a greater risk of developing depressive symptoms and suicidal ideation [39,40,41], both in girls and in boys [42]. Few studies, however, have analyzed the contribution of parents’ educational level as a factor distinct from socioeconomic status. This research, although limited, has found a negative association between parents’ educational level and the development of depressive symptoms [39] and suicidal ideation [36].

Therefore, despite the clear relationship between socioeconomic status and parents’ educational level, the available literature highlights the importance of independently studying the influence of parents’ educational level on the development of depressive symptoms and suicidal ideation in adolescents, in order to fill a gap in our knowledge that currently exists. Furthermore, most studies have focused on either a referred sample or a non-referred sample. Studies that use a combined design, in which both types of samples are integrated simultaneously, remain scarce. Thus, it seems necessary to know whether their contribution is similar or different in clinical and community adolescents. In addition, research should go one step further and analyze whether certain social and economic factors can modify the relationships that the scientific literature has shown to exist between depressive symptoms and suicidal ideation. To date, however, few studies have investigated the moderating role that such variables as adolescents’ age and gender, parents’ educational level, and family socioeconomic status play in the relationship between the two internalizing problems. These data may help us to identify vulnerable populations in order to develop intervention policies and programs for preventing or decreasing the manifestation of these two internalizing problems.

### 1.3. The Present Study

Considering the above-described background, the first objective of this study was to analyze the association between certain demographic (i.e., age and gender) and social and economic factors (i.e., mother’s education level, father’s education level, and family socioeconomic status) and depressive symptoms and suicidal ideation, in a mixed sample of referred and non-referred adolescents. Based on the existing literature, we expected to find higher levels of depressive symptoms and suicidal ideation in the group of referred adolescents, during middle and late adolescence, and among those adolescents—referred or non-referred—from families with a lower socioeconomic status and parental educational level. Regarding gender differences, we expected girls in the referred sample to exhibit higher levels of depressive symptoms and suicidal ideation than boys and that these differences, although smaller, would also be found in non-referred adolescents.

The second objective was to explore the moderating role that these demographic, social, and economic factors play in the relationship between depressive symptoms and suicidal ideation in both samples. Due to the lack of available evidence, no expectations were set for this second objective.

## 2. Materials and Methods

### 2.1. Participants and Procedure

This study used two groups of participants: a group of referred adolescents (*n* = 211) and a group of non-referred adolescents (*n* = 1401). The main demographic characteristics of the participants are summarized in Table 1.

Referred adolescents were recruited from 12 Child and Adolescent Mental Health facilities of the public health system in the southern region of Spain according to a quota sampling procedure. The procedure had two stages. First, a total of 522 potential participants satisfying the selection criteria (aged between 12 and 17 years old, living with his/her family instead of residing in an institution, not enduring a psychotic episode or outbreak, and not being intellectually disabled) were identified. To represent the diverse range of youths who use mental health facilities, we divided the potential participants into 24 mutually exclusive subgroups based on their gender (boy or girl), age (12–14 or 15–17 years old), primary referral problem (externalizing, internalizing, or other problem), and parental educational attainment (basic education (primary school, secondary school and medium vocational training) or higher education (high-school degree, high vocational training, and university)). Each of the 24 subgroups (2 × gender, 2 × age, 3 × primary referral problem, and 2 × parental educational attainment) was assigned the same number of participants (*n* = 10). Second, researchers contacted 287 randomly chosen parents to obtain consent to the participation of youths in the study. Of the 24 subgroups, 7 had less than 10 eligible participants. The acceptance rate was high (75.96%).

Non-referred adolescents were recruited from 12 high schools located in the same areas as the Mental Health facilities. High schools were selected according to the size of the municipality (<30,000 or ≥30,000 inhabitants for small and larger municipalities, respectively), the annual average per capita income of the school area (<21,966 € for low-income areas and ≥21,966 € for higher-income areas), and the type of school (public or private).

Informed consent was obtained from adolescents and parents. Data on the referred sample were collected at the clinical facilities. Data on the non-referred sample were collected in the school setting during school hours. Participation was anonymous and voluntary. Trained researchers distributed the questionnaires and assisted the participants during data collection. This study was approved by the Biomedical Research Ethics Review Board of Andalusia (Spain).

### 2.2. Measures

#### 2.2.1. Demographic, Social, and Economic Factors

An ad hoc questionnaire was used to record information about the main demographic and social characteristics of the participants, including age (12–13, 14–15, or 16–17), gender (boy or girl), and parents’ education level (no education, primary education, secondary education, or higher education).

To evaluate participants’ socioeconomic status (SES), we used the Family Affluence Scale II (FAS II) [43]. This is a brief measure of family wealth that includes four items regarding family holidays in the last 12 months, car ownership, bedroom occupancy, and home computers. The FAS II score, which ranged from 0 to 9, was computed by summing the responses to these four items. Following Currie et al. [44], this score was recoded into a three-point score for low (0–3), middle (4–6), and high (7–9) SES groups.

#### 2.2.2. Depressive Symptoms

The anxious/depressed scale of the youth self-report (YSR) [45] was used to evaluate depressive symptoms. The YSR comprises 112 items that assess a range of adolescents’ emotional and behavioral problems. The scale applied in this study includes 13 items (e.g., “I feel worthless or inferior”) answered on a Likert scale ranging from 0 (not true) to 2 (very true or often true). Cronbach’s alpha was 0.86 for the referred sample and 0.78 for the non-referred sample.

#### 2.2.3. Suicidal Ideation

Suicidal ideation was evaluated using the negative subscale of the positive and negative suicide ideation inventory (PANSI) [46,47]. This questionnaire is based on the assumption that certain factors increase the risk of suicidal behavior (e.g., negative thoughts), whereas other factors modulate or buffer against this behavior (e.g., positive thoughts). It is comprised of 14 items grouped into two subscales that evaluate the presence of protective factors and risk factors associated with suicidal thoughts during the past two weeks. Items were answered on a Likert scale ranging from 1 (none of the time) to 5 (most of the time). For the purpose of this study, we only considered the negative subscale of risk factors (8 items; e.g., “During the past two weeks, including today, how often have you thought that your problems were so overwhelming that suicide was seen as the only option for you?”). Cronbach’s alpha was 0.95 and 0.94 for the referred sample and the non-referred sample, respectively.

### 2.3. Data Analysis

Data analyses were performed using IBM SPSS Statistics (IBM Corp., Armonk, NY, USA) for MacOS, version 26. Little’s [48] missing completely at random (MCAR) test was not significant in both samples (*χ*²/df = 0.27, *p* > 0.05, for the referred sample; *χ*²/df = 2.67, *p* > 0.05, for the non-referred sample), suggesting that missing data were completely at random.

Most analyses were conducted separately for the referred and non-referred groups, since both samples were obtained through a quota sampling procedure that controlled for age, gender, parents’ educational level, and socioeconomic status to facilitate subgroups comparisons within each sample and to systematize the variations produced by the demographic, social, and economic factors of interest [49].

As preliminary analyses, we calculated correlations between the demographic, social, and economic factors and depressive symptoms and suicidal ideation.

Next, we carried out a multivariate analysis of variance (MANOVA) to determine the existence of differences in depressive symptoms and suicidal ideation between referred and non-referred samples. This analysis included mental health status (referred or non-referred) as a fixed factor and internalizing problems (i.e., depressive symptoms and suicidal ideation) as dependent variables.

Then, we conducted two factorial MANOVAs—one per mental health status group—in order to examine the effects of demographic, social, and economic factors on internalizing problems in both samples, including age range (12–13, 14–15, or 16–17), gender (boy or girl), mother’s education level (no education, primary education, secondary education, or higher education), father’s education level (no education, primary education, secondary education, or higher education), and socioeconomic status (low, medium, or high) as independent variables, and depressive symptoms and suicidal ideation as dependent variables. Complementary Bonferroni tests were carried out to evaluate multiple comparisons.

Finally, we conducted moderation analyses using the macro PROCESS 3.4 for SPSS Statistics. Five moderation models per sample were performed using depressive symptoms as an independent variable (X), suicidal ideation as a dependent variable (Y), and each demographic, social, or economic factor (i.e., age, gender, mother’s education level, father’s education level, and socioeconomic status) as a possible model moderator (W). Categorical moderators with more than two levels (i.e., age and SES) were coded as dummy variables ($W_{1}$ and $W_{2}$, respectively). All applications were based on 5000 bootstrap samples with a 95% confidence interval.

## 3. Results

### 3.1. Preliminary Analyses

Bivariate correlations for the study variables are displayed in Table 2. In both samples, mother’s education level, father’s education level, and socioeconomic status were significantly and positively correlated to each other, and socioeconomic status was negatively corelated to depressive symptoms and suicidal ideation. The correlation between depressive symptoms and suicidal ideation was strong and positive. In the referred sample, age correlated positively to depressive symptoms and suicidal ideation, and mother’s education level was negatively associated with suicidal ideation. In the non-referred sample, age and father’s education level were negatively related to suicidal ideation.

### 3.2. The Effect of Demographic, Social, and Economic Factors on Depressive Symptoms and Suicidal Ideation

Before we performed the main analysis, we conducted a MANOVA to examine differences in depressive symptoms and suicidal ideation between samples. A significant main effect was found for mental health status (Wilks’ Lambda = 0.07, *F*(2, 1556) = 55.45, *p* < 0.001, *η^2^* = 0.07). As expected, referred adolescents scored significantly higher in depressive symptoms (*F*(1, 1557) = 95.11, *p* < 0.001, *η*^2^ = 0.06 (*M* = 0.74, *SD* = 0.47 for referred adolescents; *M* = 0.49, *SD* = 0.33, for non-referred adolescents)) and suicidal ideation (*F*(1, 1557) = 78.67, *p* < 0.001, *η*^2^ = 0.05 (*M* = 1.78, *SD* = 1.15, for referred adolescents; *M* = 1.29, *SD* = 0.66, for non-referred adolescents)) than non-referred adolescents.

Then, we carried out MANOVAs in both samples to examine the effect of demographic, social, and economic factors on depressive symptoms and suicidal ideation. For the referred group, no significant multivariate effects were obtained (see Table 3). The univariate analysis only showed significant differences in terms of socioeconomic status for suicidal ideation scores (*F*(2, 28) = 4.09, *p* = 0.02, *η^2^* = 0.09). Bonferroni tests showed that adolescents from families with a low socioeconomic status scored higher in suicidal ideation than those from families with a medium or high socioeconomic status. No interaction effects were found among demographic, social, and economic factors.

Table 4 displays the MANOVA results for the non-referred group. Significant multivariate effects were obtained for age (Wilks’ Lambda = 0.01, *F*(4, 2240) = 3.17, *p* = 0.01, *η^2^* = 0.01) and socioeconomic status (Wilks’ Lambda = 0.01, *F*(4, 2240) = 2.54, *p* = 0.04, *η^2^* = 0.005). Regarding depressive symptoms, the univariate analyses showed differences in terms of age (*F*(2, 1120) = 3.67, *p* = 0.02, *η^2^* = 0.01) and father’s education level (*F*(3, 1120) = 3.26, *p* = 0.02, *η^2^* = 0.01). A post hoc analysis indicated that adolescents in the 14–15 age group showed a higher level of depressive symptoms than adolescents in the other age groups (i.e., 12–13 and 16–17). Likewise, the group of adolescents with fathers with no education scored higher in depressive symptoms than all other participants (i.e., fathers with a completed primary, secondary, or higher education). No interaction effects were observed among demographic, social, and economic factors.

Regarding suicidal ideation, the univariate analyses showed differences in terms of age (*F*(2, 1120) = 5.50, *p* = 0.004, *η^2^* = 0.01) and socioeconomic status (*F*(2, 1120) = 4.78, *p* = 0.009, *η^2^* = 0.01). Bonferroni tests showed that youths in the 14–15 year-old age group displayed higher levels of suicidal ideation than youths in the other age groups (12–13 or 16–17 years old). Also, adolescents in the low socioeconomic status group showed a higher level of suicidal ideation than adolescents in the medium and high socioeconomic status groups. We observed interaction effects between age and father’s education level (*F*(6, 1120) = 3.73, *p* < 0.001, *η^2^* = 0.02); gender, mother’s level education, and socioeconomic status *(F*(5, 1120) = 2.52, *p* = 0.03, *η^2^* = 0.01); and age, gender, father’s level education, and socioeconomic status *(F*(9, 1120) = 2.06, *p* = 0.03, *η^2^* = 0.02). Taken together, these results indicate a higher level of suicidal ideation among girls in the 14–15 year-old age group with parents with no education and whose family has a low socioeconomic status.

### 3.3. The Moderating Role That Demographic, Social, and Economic Factors Play in the Depressive Symptoms-Suicidal Ideation Relationship

**Figure 1:**
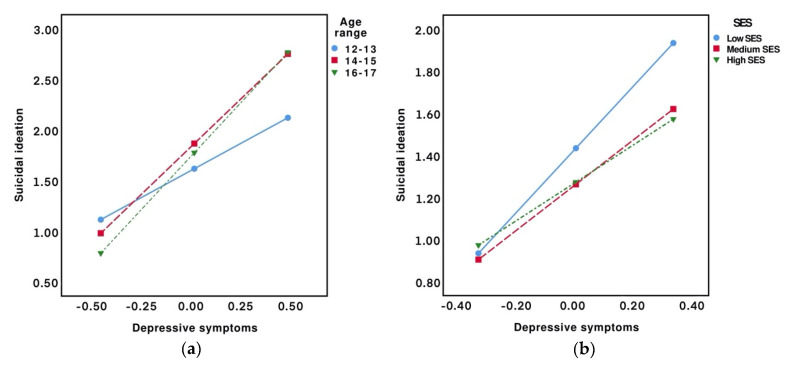
(**a**) Plot diagram for the conditional effects of depressive symptoms (−1 SD, mean, +1 SD) on suicidal ideation for the values of age range in the referred sample; (**b**) Plot diagram for the conditional effects of depressive symptoms (−1 SD, mean, +1 SD) on suicidal ideation for the values of socioeconomic status (SES) in the non-referred sample.

Moderation analyses were conducted separately for referred and non-referred adolescents to determine the individual moderation role that each demographic, social, or economic factor (W) played in the relationship between depressive symptoms (X) and suicidal ideation (Y). In the referred sample, the results only showed moderation effects in the model that included age as a moderator (*F*(2, 197) = 8.08, *p* < 0.001, ∆*R^2^* = 0.03). As expected, depressive symptoms were significantly related to suicidal ideation ($\beta_{X}$ = 1.07, *p* < 0.001, 95% CI [0.68, 1.45]). However, although age was not associated with suicidal ideation ($\beta_{W_{1}}$ = 0.25, *p* = 0.08, 95% CI [−0.03, 0.53]; $\beta_{W_{2}}$ = 0.16, *p* = 0.23, 95% CI [−0.10, 0.42]), the interaction effect of depressive symptoms and age on suicidal ideation was statistically significant ($\beta_{X*W_{1}}$ = 0.81, *p* = 0.006, 95% CI [0.23, 1.40]; $\beta_{X*W_{2}}$ = 1.04, *p* < 0.001, 95% CI [0.51, 1.57]). This indicates that the relationship between depressive symptoms and suicidal ideation was dissimilar among the three age groups. The plotted interaction effects (Figure 1a) indicate that the relationship between depressive symptoms and suicidal ideation was stronger in the 14–15 (*B* = 1.88, *p* < 0.001) and 16–17 (*B* = 2.11, *p* < 0.001) age groups than in the 12–13 age group (*B* = 1.07, *p* < 0.001).

In the non-referred sample, only socioeconomic status was found to moderate the relationship between depressive symptoms and suicidal ideation (*F*(2, 1350) = 3.06, *p* = 0.04, ∆*R^2^* = 0.01). The results showed a significant simple effect of depressive symptoms ($\beta_{X}$ = 1.53, *p* < 0.001, 95% CI [1.09, 1.97]) and socioeconomic status ($\beta_{W_{1}}$ = −0.17, *p* = 0.002, 95% CI [−0.28, −0.06]; $\beta_{W_{2}}$ = −0.16, *p* = 0.004, 95% CI [−0.27, −0.05]) on suicidal ideation. Likewise, we found a significant interaction effect of depressive symptoms and socioeconomic status on suicidal ideation ($\beta_{X*W_{1}}$ = −0.43, *p* = 0.09, 95% CI [−0.94, 0.07]; $\beta_{X*W_{2}}$ = −0.61, *p* < 0.01, 95% CI [−1.10, −0.12]). As shown in Figure 1b, this interaction effect suggests that the relationship between depressive symptoms and suicidal ideation was stronger in those adolescents whose socioeconomic status was low (*B* = 1.53, *p* < 0.001). In comparison, in adolescents with a medium (*B* = 1.10, *p* < 0.001) or high socioeconomic status (*B* = 0.92, *p* < 0.001), the strength of this association was similar.

## 4. Discussion

This study had two main objectives. The first objective was to examine the association between certain demographic, social, and economic factors and depressive symptoms and suicidal ideation in referred and non-referred adolescents. The second objective was to explore the moderating effect of these factors on the relationship between depressive symptoms and suicidal ideation in both groups.

As expected, referred adolescents showed higher levels of depressive symptoms and suicidal ideation than non-referred adolescents. In addition, regarding age, we expected to find higher levels of internalizing problems in middle and late adolescence in both groups. As highlighted in the introduction, most studies found an increase in the levels of depressive symptoms and suicidal ideation throughout adolescence [5,6], although some studies have reported stability in these levels [16,17]. Our findings in referred adolescents showed stable levels of depressive symptoms and suicidal ideation during adolescence. In comparison, in non-referred adolescents, our data indicate an increase in the levels of depressive symptoms and suicidal ideation between 12–13 and 14–15 years old, and stability from 14–15 to 16–17 years old. These results suggest different developmental trends of internalizing problems among referred and non-referred adolescents. However, the cross-sectional nature of our data precludes the establishment of causality. Further longitudinal studies are required to clarify these trends.

Another interesting finding is that when both socioeconomic status and parents’ educational level were considered in the same analysis, suicidal ideation was significantly associated with socioeconomic status, but not with parents’ educational level. As predicted, and consistent with available evidence [40,41], we found higher levels of suicidal ideation among referred and non-referred adolescents from families with a low socioeconomic status. In contrast, the relationship between socioeconomic status and depressive symptoms was not significant when parents’ educational level was partialled out. In line with previous research [39], our results also evidenced a significant and negative relationship between father’s educational level and the development of depressive symptoms.

The above findings support the convenience of analyzing the effect of socioeconomic status and parents’ educational level based on differentiated variables. In addition, we must emphasize that our data indicate that socioeconomic status is related to suicidal ideation, in both referred and non-referred adolescents, but not to depressive symptoms. A possible explanation for this finding could be related to the factors that are involved in the increase of both problems during adolescence. While the development of depressive symptoms could be associated with the physical and psychological changes that come along with puberty [50], an increase in suicidal ideation may be linked to the occurrence of other mental health problems [34,51]. In view of this, socioeconomic status could be relevant to suicidal ideation only, and not to depressive symptoms, because suicidal ideation is more strongly associated with access to mental health services than depressive symptoms. Thus, belonging to a family with a low socioeconomic status, which limits access to resources for mental health promotion [40], may result in additional psychological problems and, consequently, increase the risk of developing higher levels of suicidal ideation. As opposed to our expectations, a low socioeconomic status does not seem to be a determining factor for depressive symptoms, perhaps because these symptoms are related to normative changes in adolescence [32] that do not always require specialized mental health care.

The role that parents’ educational level plays in depressive symptoms may be explained by previous studies showing that fathers with a higher educational level tend to be warmer and more communicative with their children and to possess better emotional skills [52,53]. These aspects may be, in turn, associated with lower levels of depressive symptoms. In fact, warmth and supportive parenting have been postulated to be protective factors against the development of depressive symptoms during adolescence [54,55].

Finally, regarding the first objective, we found no gender differences in the levels of depressive symptoms and suicidal ideation among referred and non-referred adolescents. Although the absence of gender differences in a non-referred sample has been observed in prior studies [23,24,25], most studies with referred samples have found higher levels of depressive symptoms and suicidal ideation among girls belonging to this group [21,22]. A possible explanation for our findings could be the reduction of differences in sex-type roles adopted by adolescent boys and girls [56]. Although our study did not confirm a direct effect of gender on the levels of depressive symptoms and suicidal ideation in referred and non-referred adolescents, we did find an interaction effect between the gender and age of adolescents, parents’ educational level, and family socioeconomic status in the non-referred sample. In this adolescent group, girls who were 14–15 years old, had parents with no education, and belonged to families with a low socioeconomic status reported higher levels of suicidal ideation. These results suggest that, in addition to analyzing gender and age in the development of depressive symptoms and suicidal ideation, it is necessary to consider the effect of certain social and economic factors that may contribute to the identification of vulnerable groups that could be target populations of preventive programs.

Regarding the second objective, we found a positive and strong association between depressive symptoms and suicidal ideation in both groups. This relationship, which was consistent with previous studies [28], was also found to be moderated by different factors.

In the referred sample, this link was influenced by adolescents’ age. Specifically, our results showed that the relationship between depressive symptoms and suicidal ideation was stronger in the 14–15 and 16–17 year-old age groups, and weaker in early adolescence (the 12–13 year-old age group). These results suggest that this relationship becomes stronger as adolescence progresses. A possible explanation could be related to the stages of cognitive development during adolescence and the acquisition of formal operational in middle adolescence, that is at 14–15 years old [57]. Boys and girls in mid–late adolescence have greater cognitive abilities and exhibit more complex thinking [58]. This may allow them to reflect and consider suicide to be an easy way out of a depressive situation, which in the referred sample sharpens significantly with age. Although adolescents in the 12–13 year-old age group do experience depressive symptoms, they may not have reached a sufficient level of cognitive maturation for the elaboration of a hypothetical plan to commit suicide or to think of suicide as a possible solution to their problems.

In the non-referred sample, our results indicated that it was not age, but family socioeconomic status that moderated the relationship between depressive symptoms and suicidal ideation. Our results showed that the association between depressive symptoms and suicidal ideation was stronger in adolescents with a low socioeconomic status. These findings, in line with previous research [39,40,41], indicate that a low family socioeconomic status may be a risk factor for the emergence and maintenance of some internalizing problems. These results may help us to identify vulnerable populations and evidence the convenience of developing primary prevention programs designed to target non-referred adolescents who come from economically disadvantaged family contexts. In this context, it may be beneficial to implement in school programs that promote mental health. These programs could promote the competences and well-being that prevent the occurrence of internalizing problems in adolescents [59].

Although the present study yielded some interesting findings, these should be considered in light of the study’s limitations. Firstly, this study had a cross-sectional design which allows us to examine the association between variables, but not to make inferences about causality. Further longitudinal studies are required to shed light on the directionality of relationships and to analyze the developmental trajectory of depressive symptoms and suicidal ideation in referred and non-referred adolescents. Second, assessments of depressive symptoms and suicidal ideation, as well as of social and economic factors, were based on adolescents’ self-reports, which may have artificially inflated the strength of the observed associations through shared method variance. However, some authors, such as Andrew et al. [60], have found that adolescents report more realistically about their internal states than their parents or other external informants. Third, we developed this study by considering two samples that were obtained by a quota sampling procedure that controls for the variables age, gender, parents’ educational level, and socioeconomic status. This type of sampling procedure was chosen to facilitate comparisons between subgroups and the systematization of the variations produced by the sociodemographic factors in each sample [49]. Nevertheless, this sampling procedure prevented us from offering prevalence rates and comparing samples in terms of some social and economic factors because their presence was controlled based on the established quotas. Finally, it is important that future research examines other social and economic factors intimately related to depressive symptoms and suicidal ideation, such as access to mental health services. The study of a more diverse variety of demographic, social, and economic factors could provide a more complete picture of the factors involved in the development of internalizing problems during adolescence.

## 5. Conclusions

The findings of this study emphasize the importance of examining the role that certain demographic, social, and economic factors play in the development of depressive symptoms and suicidal ideation during adolescence. This study also highlights the utility of employing a combined design in which referred and non-referred adolescents are assessed simultaneously. Our results suggest that it is possible that there is no single unique developmental trajectory for these internalizing problems, but a differentiated trend that is based on the individual’s mental health status. Moreover, we did not find gender differences in depressive symptoms and suicidal ideation in referred and non-referred adolescents. However, the interaction effect demonstrated that, in non-referred adolescents, suicidal ideation levels were higher in girls who were 14–15 years old, with parents without studies, and belonging to families with a low socioeconomic status. These results emphasized the importance of analyzing, together with gender and age, other social and economic factors associated with the development of depressive symptoms and suicidal ideation.

In addition, our findings evidence the convenience of considering socioeconomic status and parents’ educational level as differentiated variables. When both variables were studied together, it was found that socioeconomic status was related to suicidal ideation and that parental educational level was associated with depressive symptoms.

Finally, our data show that demographic, social, and economic factors moderate the relationship between depressive symptoms and suicidal ideation. In the referred sample, our results show that the relationship between depressive symptoms and suicidal ideation was moderated by age and becomes stronger as adolescence progresses. In the non-referred sample, it was observed that family socioeconomic status moderated this relationship, finding that this relationship was stronger in those adolescents whose family socioeconomic status was low. As such, it is important to consider the moderating effect of these factors on the relationship between depressive symptoms and suicidal ideation with the objective of developing specific prevention strategies for each adolescent group.

## Figures and Tables

**Table 1:** Main demographic characteristics of the referred (*n* = 211) and the non-referred sample (*n* = 1401).

Variables	Referred (*n* = 211)	Non-Referred (*n* = 1401)
Gender, *n* (%)		
Female	120 (56.9%)	744 (53.1%)
Male	91 (43.1%)	657 (46.9%)
Age, mean (*SD*)	14.44 (1.64)	14.79 (1.54)
Education Level, *n* (%)		
Compulsory Secondary Education	171 (81%)	1062 (75.8%)
Post-Compulsory Education	20 (9.5%)	288 (20.6%)
Vocational Training	20 (9.5%)	51 (3.6%)
Family Structure, *n* (%)		
Two-parent families	148 (70.1%)	1156 (82.3%)
Single-parent families	36 (17.1%)	134 (9.6%)
Reconstituted families	21 (10%)	88 (6.3%)
Lived with other relatives	6 (2.8%)	19 (1.4%)
Institutionalized homes	-	4 (0.4%)

**Table 2:** Correlations among the demographic, social, and economic factors and internalizing problems for the referred (*n* = 211) and the non-referred sample (*n* = 1401).

	AGE	MEL	FEL	SES	DS	SI
**Referred sample (*n* = 211)**						
Age	-	-	-	-	-	-
Mother’s Education Level (MEL)	−0.10	-	-	-	-	-
Father’s Education Level (FEL)	−0.10	0.45 ***	-	-	-	-
Socioeconomic Status (SES)	−0.07	0.21 **	0.23 **	-	-	-
Depressive Symptoms (DS)	0.15 *	−0.10	0.02	−0.17 *	-	-
Suicidal Ideation (SI)	0.15 *	−0.17 *	−0.14	−0.17 *	0.70 ***	-
**Non-referred sample (*n* = 1401)**						
Age	-	-	-	-	-	-
Mother’s Education Level (MEL)	−0.03	-	-	-	-	-
Father’s Education Level (FEL)	−0.04	0.58 ***	-	-	-	-
Socioeconomic Status (SES)	−0.01	0.30 ***	0.31 ***	-	-	-
Depressive Symptoms (DS)	0.02	−0.01	−0.03	−0.08 **	-	-
Suicidal Ideation (SI)	−0.08 **	−0.04	−0.08 **	−0.10 ***	0.53 **	-

Note: * *p* < 0.05; ** *p* < 0.01; *** *p* < 0.001.

**Table 3:** Multivariate analysis of variance (MANOVA) for the referred sample (*n* = 211).

Demographic, Social, and Economic Factors	Depressive Symptoms		Suicidal Ideation	
	*M* (*SD*)	*F/η^2^*	*M* (*SD*)	*F/η^2^*
Age		0.64/0.02		1.83/0.05
12–13	0.66 (0.45)		1.53 (0.88)	
14–15	0.75 (0.47)		1.88 (1.27)	
16–17	0.83 (0.47)		1.96 (1.24)	
Gender		1.28/0.02		1.08/0.01
Boy	0.63 (0.45)		1.50 (0.93)	
Girl	0.83 (0.47)		2.00 (1.26)	
Mother’s education level		0.72/0.03		1.17/0.04
No education	1.05 (0.45)		2.53 (1.35)	
Primary education	0.75 (0.51)		1.93 (1.23)	
Secondary education	0.66 (0.43)		1.47 (0.98)	
Higher education	0.76 (0.44)		1.79 (1.10)	
Father’s education level		1.42/0.05		0.89/0.03
No education	0.75 (0.51)		2.07 (1.26)	
Primary education	0.74 (0.47)		1.86 (1.19)	
Secondary education	0.76 (0.48)		1.73 (1.15)	
Higher education	0.75 (0.47)		1.47 (0.97)	
Socioeconomic status (SES)		1.74/0.04		4.08 */0.10
Low	0.89 (0.49)		2.05 (1.28)	
Medium	0.72 (0.48)		1.63 (1.19)	
High	0.65 (0.40)		1.48 (0.87)	

Note: * *p* < 0.05.

**Table 4:** Multivariate analysis of variance (MANOVA) for the non-referred sample (*n* = 1401).

Demographic, Social, and Economic Factors	Depressive Symptoms		Suicidal Ideation	
	*M* (*SD*)	*F/η^2^*	*M* (*SD*)	*F/η^2^*
Age		3.67 */0.01		5.50 */0.01
12–13	0.45 (0.33)		1.32 (0.71)	
14–15	0.52 (0.34)		1.35 (0.74)	
16–17	0.48 (0.31)		1.21 (0.52)	
Gender		1.24/0.001		0.37/0.001
Boy	0.41 (0.30)		1.24 (0.57)	
Girl	0.55 (0.33)		1.33 (0.72)	
Mother’s education level		0.69/0.002		1.29/0.003
No education	0.44 (0.28)		1.29 (0.66)	
Primary education	0.51 (0.34)		1.32 (0.72)	
Secondary education	0.49 (0.33)		1.27 (0.63)	
Higher education	0.47 (0.31)		1.27 (0.61)	
Father’s education level		3.26 */0.01		1.28/0.003
No education	0.56 (0.33)		1.47 (0.84)	
Primary education	0.48 (0.32)		1.31 (0.71)	
Secondary education	0.47 (0.32)		1.22 (0.53)	
Higher education	0.48 (0.32)		1.26 (0.62)	
Socioeconomic status (SES)		1.51/0.003		4.78 **/0.01
Low	0.55 (0.35)		1.53 (0.87)	
Medium	0.50 (0.32)		1.27 (0.65)	
High	0.46 (0.32)		1.25 (0.60)	

Note: * *p* < 0.05; ** *p* < 0.01.
